# Uptake of HIV testing and counseling, risk perception and linkage to HIV care among Thai university students

**DOI:** 10.1186/s12889-016-3274-8

**Published:** 2016-07-12

**Authors:** Thana Khawcharoenporn, Krongtip Chunloy, Anucha Apisarnthanarak

**Affiliations:** Division of Infectious Diseases, Faculty of Medicine, Thammasat University, Pathumthani, Thailand; HIV/AIDS Care Unit of Thammasat Univesity Hospital, Pathumthani, Thailand

**Keywords:** Human immunodeficiency virus, Testing and counseling, Risk perception, Linkage to care, University students, Thailand

## Abstract

**Background:**

HIV testing and counseling (HTC) with linkage to care after known infection are key components for HIV transmission prevention. This study was conducted to assess HTC uptake, HIV risk perception and linkage to care among Thai university students.

**Methods:**

An outreach HTC program was conducted in a large public university in Thailand from January 2013 to December 2014. The program consisted of brief HIV knowledge assessment, free HTC, HIV risk assessment and education provided by the healthcare personnel. Students were categorized into low, moderate and high-risk groups according to the pre-defined HIV risk characteristics.

**Results:**

One-thousand-eight-hundred-one students participated in the program, 494 (27 %) underwent HTC. Independent characteristics associated with no HTC uptake included female sex (*P* < 0.001), lower HIV knowledge score (*P* < 0.001), younger age (*P* < 0.001) and students from non-health science faculties (*P* = 0.02). Among the 494 students undergoing HTC, 141 (29 %) were categorized into moderate or high-risk group, of whom 45/141 (32 %) had false perception of low HIV risk. Being heterosexual was independently associated with false perception of low HIV risk (*P* = 0.04). The rate of new HIV infection diagnosis was 4/494 (0.8 %). Of these 4 HIV-infected students, 3 (75 %) were men who have sex with men and only 2 of the 4 students (50 %) showed up for HIV continuity care.

**Conclusions:**

An outreach HIV prevention program with HTC was feasible and beneficial in detecting HIV risk and infection among the university students. However, interventions to improve HTC uptake, HIV risk perception and linkage to care are needed.

## Background

Human immunodeficiency virus (HIV) infection and acquired immune deficiency syndrome (AIDS) have been major public health problems in Thailand with the prevalence of HIV infection of 1.1 % and 8200 newly-diagnosed HIV-infected individuals in 2013 [1]. The highest number of AIDS cases was observed in the age group of 30–34 years (24.97 %) followed by the age group of 25–29 years (21.73 %) [2]. Given the lag period of 5–15 years between the estimated time of acquiring HIV and the time of AIDS diagnosis observed among Thai HIV-infected patients at our hospital, individuals in adolescence and early adulthood may have high rates of HIV acquisition in Thailand.

University students are amongst populations in the age groups with the highest probability of acquiring HIV. Our previous study conducted among university students indicated that despite their high level of knowledge, HIV risks and risk behaviors were commonly reported and some of the students were not aware of their HIV risks [3]. Nonetheless, uptake of HIV testing and counseling (HTC), the impact of HIV risk perception on HTC acceptance and linkage to care after diagnosis of HIV infection have not been evaluated in Thai university students.

Universal HTC has been recommended in the communities where HIV prevalence is 0.1 % or higher to increase awareness of HIV status, HIV diagnosis and linkage of HIV-infected persons to care, and prevent HIV transmission [4]. During the HTC program, HIV risk perception should be assessed and targeted education to improve risk perception and reduce risk behaviors should be provided. In Thailand, HIV testing is available free of charge and can be done twice a year, as a part of an HIV prevention campaign conducted by The Ministry of Public Health. However, specific guidelines and protocols regarding how to implement HTC in key populations, especially adolescence and young adults have not been recommended. We conducted this study to evaluate the feasibility of an outreach HIV prevention program that incorporates HTC and linkage to care after an HIV positive test result. In addition, this study aimed to assess HIV risks, risk behaviors and HIV risk perception and its impact on HTC uptake and to determine the rate of and factors associated with HIV infection among Thai university students.

## Methods

### Study population, setting and design

This is a prospective study conducted between 1st January 2013 and 31st December 2014 among Thai students from six different faculties of a large public university, including Science, Engineering, Medicine, Medical Technology, Journalism and Mass Communication, and Laws. These 6 faculties were chosen as they are representative of university students from major faculties in medical science, science, and social science fields. This study was conducted in accordance with the amended Declaration of Helsinki and was approved by the Faculty of Medicine, Thammasat University Ethics Committee.

### Study protocol

An outreach HIV prevention program that incorporated HTC was conducted during the study period by the HIV care team of Thammasat University Hospital. This team consists of 3 Infectious Diseases doctors, 2 HIV specialist nurses, 2 non-physician medical assistants and 2 HIV-infected volunteers, to promote HIV prevention and provide relevant education for the students. The program was conducted in designated open-space areas of the main building of each faculty from 10.00 a.m. to 2.00 p.m. weekly. Private rooms for HTC were available within the designated areas. Students in each faculty were approached and asked to participate in the program. Brief HIV knowledge assessment questionnaire, free HTC and education on HIV transmission prevention by the HIV care team were provided in the program. The brief questionnaire asked the participants to answer whether the 12 different statements about HIV transmission were true, false or they did not know the answer. HIV knowledge score was calculated based on the number of correct answer to these 12 statements. Demographics data, HTC acceptance and reasons for accepting or declining HTC were collected in the brief questionnaire form.

The students who voluntarily accepted HTC underwent pre-test counseling conducted by the HIV counselors. Data about demographics, HIV acquisition risks and risk behaviors, and HIV risk perception of the students were collected using the counseling form. The questions in the counseling form were derived from the survey form validated in previous studies [3, 5]. The students who reported history of HIV infection were excluded from the HIV testing. A anti-HIV blood test was performed after the counseling. Primary and secondary telephone numbers of each student were recorded by the counselors. These contact numbers were used for HIV test result notification.

### Study definitions

Sexual orientation and HIV risk perception were self-identified by the students. The students identified their own HIV risks by answering “No risk at all”, “A little risk (low-risk)”, “More than a little (moderate-risk)” and “A lot of risk (high-risk)” to the HIV counselors. Actual HIV risk of the students was defined as “low-risk”, “moderate-risk” and “high-risk” based on the pre-specified risk characteristics and risk behaviors according to previous studies’ criteria (Table 1) [3, 5]. Students who were categorized as moderate or high-risk but perceived their risks as no or low-risk were classified as having false perception of low HIV risk.

**Table 1:** Human immunodeficiency virus (HIV) risk stratification according to the pre-specified reported characteristics and behaviors of the students

Characteristics and behaviors	HIV risk		
	Low	Moderate	High
Number of different sexual partners within 30 days			
0–1	√		
2–3		√	
> 3			√
Number of new sexual partners within 30 days			
0–1	√		
2–3		√	
> 3			√
Using condom with vaginal sex			
Always	√		
Most of the time	√		
About a half of time		√	
Sometimes			√
Never			√
Using condom with oral sex			
Always	√		
Most of the time	√		
About a half of time		√	
Sometimes		√	
Never		√	
Using condom with anal sex			
Always	√		
Most of the time	√		
About a half of time		√	
Sometimes			√
Never			√
Exchanging sex for money or drugs			
No	√		
Yes			√
Ever injected drug with needles			
No	√		
Yes			√
Ever shared needle to inject drugs			
Never	√		
Sometimes			√
About a half of time			√
Most of the time			√
Always			√
History of STIs within the past year			
No	√		
Yes			√
Sexual partner had STIs within the past year			
No	√		
Yes			√
Sexual partner had exchanged sex for money or drugs within 30 days			
No	√		
Yes			√
Sexual partner had used drug within 30 days			
No	√		
Yes			√

NOTE

*STIs* sexually-transmitted infections

### HIV testing result notification

Notification of the test results was done via telephone within 72 h. The HIV counselors called all students to inform the negative results. However, if the test result was positive, they would inform the students to come to the hospital for post-test counseling and result notification, in accordance with Thai laws. A total of 3 calls (1 week apart) were attempted to notify the students’ results and addition 3-week duration was allowed after that for the students to call back before contact failure was considered. Plans for their HIV continuity care were discussed with the HIV-infected students. The counselors called the students every 1 month after notification of the test results and asked whether the participants had already initiated HIV care and when they did so. If the participants had not initiated HIV care, the HIV counselors would assess and identify any problems/obstacles faced by the HIV-positive participants every time they did the follow-up calls and help to alleviate those problems/obstacles and further support the referral for care.

### Data analyses

All statistical analyses were performed using SPSS version 15.0 (SPSS, Chicago, Illinois). Categorical variables were compared using Pearson’s *χ*^2^ or Fisher’s exact test as appropriate. Continuous variables were compared using Mann Whitney *U* test. All *P* values were 2 tailed; *P* values less than .05 were considered statistically significant. Variables that were present at a significance level of *P* < .20 in univariable analysis of factors associated with no HTC acceptance, false perception of low HIV risk, and HIV infection were entered into logistic regression models. These variables were subsequently removed from the models in backward stepwise fashion if their *P* values were >0.05 until the final model had reached. Adjusted odd ratios (aORs) and 95 % confidence intervals (CIs) were determined for independent risk factors associated with no HTC acceptance, false perception of low HIV risk, and HIV infection.

## Results

### Characteristics of the study participants and HIV transmission knowledge

**Fig. 1:**
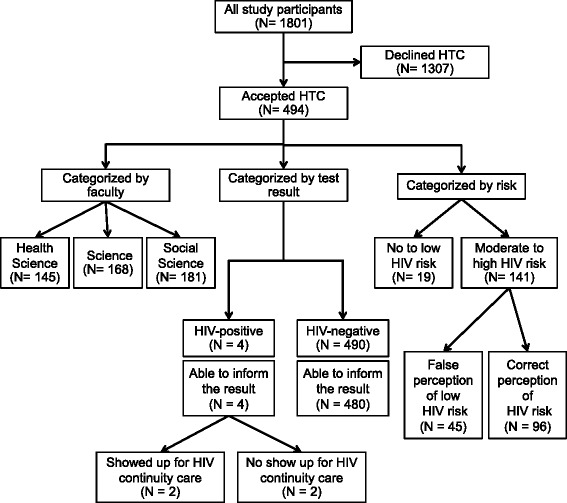
Study flow of the 1801 participating students

A total of 2250 university students from 6 different faculties were approached, of which 1801 students (80 %) agreed to participate in the HIV prevention outreach program (Fig. 1). Demographic characteristics of the 1801 students are shown in Table 2. The mean age was 20.12 years (range 17–35 years). About a half of the students were female and the majority of them were from faculties in the Social Science field. The median score for HIV transmission knowledge was 10.64 (range 4–12). More than 80 % of the students correctly stated in the survey that “AIDS is caused by a virus”, “You can get HIV from sexual contact with an HIV-infected individual”, “Mosquitoes cannot transmit HIV”, “Condon use can prevent HIV”, “You cannot get HIV from having meal with an HIV-infected individual”, “HIV-infected individuals may not have symptoms for several years”. “Asymptomatic HIV-infected individual can transmit HIV to other people”, “Persons who look clean and healthy can already be infected with HIV”, “One can know his/her HIV status by getting HIV blood test” and “Anti-HIV drugs can increase lifespan of the infected individual”. Less than 80 % of the students could correctly state that “There is currently no vaccine that can prevent HIV infection” and “Having multiple sexual partners increases risk for getting HIV”.

**Table 2:** Characteristics of the participating students stratified by HIV testing and counseling (HTC) acceptance

Characteristics	All (*N* = 1801)	Accepted HTC (*N* = 494)	Declined HTC (*N* = 1307)	*P* ^*a*^
Demographics				
Sex				**<0.001**
Male	862 (48)	277 (56)	585 (45)	
Female	922 (51)	215 (44)	707 (54)	
Transgender	17 (1)	2 (0.4)	15 (1)	
Age (years, mean, range)	20.12 (17–35)	20.48 (17–38)	19.98 (17–27)	**<0.001**
Faculty				
Health Science^b^	426 (24)	145 (29)	281 (22)	**<0.001**
Science^c^	603 (34)	168 (34)	435 (33)	
Social Science^d^	772 (43)	181 (37)	591 (45)	
Monthly household income ($US, mean, range)	2368 (30–90,909)	2416 (121–90,909)	2350 (30–30,000)	0.71
HIV transmission knowledge				
HIV transmission knowledge score^e^ (mean, range)	10.64 (4–12)	10.91 (6–12)	10.54 (4–12)	**<0.001**
Answer for the following statements AIDS is caused by a virus				**0.03**
True	1752 (97)	487 (99)	1265 (97)	
False	49 (3)	7 (1)	42 (3)	
You can get HIV from sexual contact with an HIV-infected individual				**0.04**
True	1763 (98)	489 (99)	1274 (98)	
False	38 (2)	5 (1)	33 (2)	
Mosquitoes can transmit HIV				**<0.001**
True	323 (18)	53 (11)	270 (21)	
False	1448 (82)	441 (89)	1037 (79)	
Condom use can prevent HIV infection				**0.001**
True	1703 (95)	481 (97)	1222 (94)	
False	98 (5)	13 (3)	85 (6)	
You can get HIV from having meal with an HIV-infected individual				**0.001**
True	253 (14)	48 (10)	205 (16)	
False	1548 (86)	446 (90)	1102 (84)	
HIV-infected individuals may not have symptoms for several years				**0.04**
True	1721 (96)	480 (97)	1241 (95)	
False	80 (4)	14 (3)	66 (5)	
Asymptomatic HIV-infected individual can transmit HIV to other people				**0.006**
True	1722 (96)	483 (98)	1239 (95)	
False	79 (4)	11 (2)	68 (5)	
Persons who look clean and healthy can already be infected with HIV				0.06
True	1741 (97)	484 (98)	1257 (96)	
False	60 (3)	10 (2)	50 (4)	
One can know his/her HIV infection status by getting HIV blood test				**0.002**
True	1562 (87)	448 (91)	1114 (85)	
False	239 (13)	46 (9)	193 (15)	
There is currently a vaccine that can prevent HIV infection				**0.005**
True	413 (23)	91 (18)	322 (25)	
False	1388 (77)	403 (82)	985 (75)	
Anti-HIV drugs can increase lifespan of the infected individuals				**<0.001**
True	1507 (84)	444 (90)	1063 (81)	
False	294 (16)	50 (10)	244 (19)	
Having multiple sexual partners increases risk for getting HIV				**<0.001**
True	1285 (71)	370 (75)	810 (62)	
False	516 (29)	188 (25)	497 (38)	

NOTE

Data are in numbers (%) unless otherwise indicated

^a^Comparison between students who accepted and declined UVCT

^b^Included Faculty of Medicine and Faculty of Medical Technology

^c^Included Faculty of Science and Faculty of Engineering

^d^Included Faculty of Laws and Faculty of Journalism and Mass Communication

^e^Based on number of correct answers to 12 statements about HIV transmission

*AIDS* acquired immune deficiency syndrome

The *P* values in bold represent statistical significance

### Reasons and characteristics associated with declining HTC

Among the 1801 students participating in the HIV prevention program, 494 (27 %) accepted HTC. Of the 1307 students who declined HTC, the three most common reasons were not perceiving themselves to be at risk for HIV acquisition [370 (36 %)], being afraid of blood/needle/pain [283 (28 %)] and time constraints [231 (23 %)]. Other reasons included being afraid of having a positive HIV test result, having a recent HIV test and no interest in knowing the HIV status. When comparing between students who accepted and declined HTC (Table 2), those who declined HTC were more-likely to be female, younger and from faculties in the Social Sciences field. The students who declined HTC were less likely to correctly answer the HIV knowledge items, except for the statement “Persons who look clean and healthy can already be infected with HIV”. In the multivariable logistic regression analysis, characteristics of the students independently associated with declining HTC included being female (aOR 1.55; 95 % CI 1.25–1.92; *P* < 0.001), lower knowledge score (aOR 1.26; 95 % CI 1.15–1.39; *P* < 0.001 for each score lower), younger age (aOR 1.24; 95 % CI 1.15–1.34; *P* < 0.001 for each year younger) and being a student from non-health science faculties (aOR 1.35; 95 % CI 1.05–1.72; *P* = 0.02).

### Characteristics and HIV risks among participants undergoing HTC

Of the 494 students undergoing HTC, 277 (56 %) were male and 145 (29 %), 168 (34 %) and 181 (37 %) were from faculties in the Health Science field, Science field and Social Science field, respectively. Forty-eight students (10 %) had prior negative HIV tests. The majority of the 494 participants were heterosexual [416 (84 %)] and 202 (41 %) had had sexual intercourse. Among the 202 students ever had sexual intercourse, 147 (73 %) reported having vaginal sex, 104 (51 %) reported having oral sex and 90 (45 %) reported having anal sex. The reported rates of consistent condom use were all low with each type of sex (40 % with vaginal sex, 34 % with anal sex and 18 % with oral sex). Other reported risks for HIV acquisition included having tattoo or piercing (37 %) while having sexually-transmitted infections (STIs) within the past year, having sexual partner who exchanged sex for money or drugs within the past 30 days, injection drug use and having sexual partner who had STIs within the past year were rarely reported (Table 3). Of these 494 students, 484 (98 %) were reachable for HIV result notification within the median time of 3 days (range 2–44 days) while 10 (2 %) had contact failure. Among the 10 students with contact failure, the median age was 22 years (range 19–26 years), 5 (50 %) were male, 7 (70 %) were from faculties in the Health Science field, 3 (30 %) were from faculties in the Social Science field, 1 (10 %) had prior HIV test, 10 (100 %) were heterosexual, 2 (20 %) were categorized into high-HIV-risk group and none had false perception of low HIV risk. When comparing characteristics between students from different faculties (Table 3), students from faculties in the Health Science field were older, had higher score for HIV transmission knowledge and more likely to have prior HIV test. Higher proportion of students in the Health Science and Social Science fields reported to be homosexual compared to those in the Science field. Health Science students were more likely to perceive their HIV risk higher than their actual risk while Science and Social Science students were more likely to perceive their risk lower than actual risk. However, the proportions of students who had false perception of low HIV risk were not significantly different between students in the three groups.

**Table 3:** Characteristics of the 494 students undergoing HIV testing and counseling stratified by faculty

Characteristics	Health Science^a^ (*N* = 145)	Science^b^ (*N* = 168)	Social Science^c^ (*N* = 181)	*P* ^*d*^
Demographics				
Sex				0.06
Male	74 (51)	107 (64)	96 (53)	
Female	71 (49)	61 (36)	83 (46)	
Transgender	0 (0)	0 (0)	2 (1)	
Age (years, median, range)	21 (18–35)	20 (17–28)	20 (18–26)	**<0.001**
Monthly household income ($US, median, range)	1515 (242–15,151)	1515 (152–90,909)	1818 (121–15,151)	0.19
HIV transmission knowledge score^e^ (median, range)	12 (9–12)	11 (6–12)	11 (8–12)	**<0.001**
HIV risk charactertistics				
Prior HIV test	20 (14)	9 (5)	19 (11)	**0.04**
Number of prior HIV test (median, range) Ever had sexual intercourse	1 (1–6) 48 (33)	1 (1–2) 74 (44)	1 (1–3) 80 (44)	0.21 0.08
Sexual orientation				**0.006**
Heterosexual	121 (83)	154 (92)	141 (78)	
Homosexual	22 (15)	11 (7)	32 (18)	
Bisexual	2 (1)	3 (2)	8 (4)	
Number of different sexual partner within the past 3 months (median, range)	1 (0–7)	1 (0–10)	1 (0–14)	0.49
Number of new sexual partner within the past 3 months (median, range)	0 (0–7)	0 (0–8)	0 (0–11)	0.09
Using condom with vaginal sex consistently^f^	15/31 (48)	21/66 (32)	22/50 (44)	0.21
Using condom with anal sex consistently^f^	10/24 (42)	7/21 (33)	14/45 (31)	0.68
Using condom with oral sex consistently^f^	6/29 (21)	4/30 (13)	9/45 (20)	0.71
Time since last unprotected sex (months, median, range)	4 (1–48)	1 (1–72)	3 (1–72)	0.37
Ever injected drug with needle	0 (0)	0 (0)	3 (2)	0.07
Ever had tattoo or piercing	54 (37)	57 (34)	73 (40)	0.47
Ever exchanged sex for money or drugs	0 (0)	0 (0)	0 (0)	1.00
Having STIs within the past year				**0.03**
Yes	1 (1)	0 (0)	2 (1)	
Not sure	6 (4)	18 (11)	4 (2)	
Sexual partner had STIs within the past year				0.24
Yes	1 (1)	0 (0)	0 (0)	
Not sure	9 (6)	21 (13)	15 (8)	
Sexual partner exchanged sex for money or drugs within the past 30 months				0.73
Yes	0 (0)	1 (0.6)	1 (0.6)	
Not sure	6 (4)	14 (8)	10 (6)	
Sexual partner injected drug with needle within the past 3 months				0.23
Yes	0 (0)	0 (0)	0 (0)	
Not sure	5 (3)	14 (8)	8 (4)	
Self-perception of HIV risk				**0.002**
No to low risk	84 (58)	124 (74)	134 (74)	
Moderate to high risk	61 (42)	44 (26)	47 (26)	
Actual HIV risk				**0.03**
No to low risk	116 (80)	115 (69)	122 (67)	
Moderate to high risk	29 (20)	53 (31)	59 (33)	
False perception of low HIV risk	8 (6)	17 (10)	20 (11)	0.19

NOTE

Data are in numbers (%) unless otherwise indicated

^a^Included Faculty of Medicine and Faculty of Medical Technology

^b^Included Faculty of Science and Faculty of Engineering

^c^Included Faculty of Laws and Faculty of Journalism and Mass Communication

^d^Comparison between students from health science, science and social science faculties

^e^Based on number of correct answers to 12 statements about HIV transmission

^f^The denominators were those who had each type of sexual activity

*STI* sexually-transmitted infection

The *P* values in bold represent statistical significance

### HIV risk perception and characteristics associated with false perception of low HIV risk

Among the 494 students undergoing HTC, 141 (29 %) were categorized into moderate or high-risk group, of whom 45/141 (32 %) had false perception of low HIV risk. Characteristics of students with false perception of low HIV risk and those with correct perception of risk were compared in Table 4. In the multivariable logistic regression analysis, being heterosexual was independently associated with false perception of HIV risk (aOR 2.23; 95 % CI 1.03–4.84: *P* = 0.04).

**Table 4:** Characteristics of the 141 students with moderate to high HIV risk categorized by HIV risk perception

Characteristics	All (*N* = 141)	False perception of low risk (*N* = 45)	Correct perception of risk (*N* = 96)	*P* ^*a*^
Demographics				0.20
Sex	100 (71)	28 (62)	72 (75)	
Male	40 (28)	17 (38)	23 (24)	
Female	1 (1)	0 (0)	1 (1)	
Transgender				
Age (years, median, range)	21 (18–31)	21 (18–25)	21 (18–31)	0.78
Faculty				0.26
Health Science^b^	29 (21)	8 (18)	21 (22)	
Science^c^	53 (38)	17 (38)	36 (38)	
Social Science^d^	59 (42)	20 (44)	39 (41)	
Monthly household income ($US, median, range)	1515 (121–90,909)	1515 (242–15,151)	1515 (121–90,909)	0.26
HIV transmission knowledge score^e^ (median, range)	11 (7–12)	11 (8–12)	11 (7–12)	0.82
HIV risk charactertistics				
Prior HIV test	23 (16)	4 (9)	19 (20)	0.14
Number of prior HIV test (median, range)	1 (1–6)	1 (1–6)	1 (1–6)	0.80
Ever had sexual intercourse	141 (100)	45 (100)	96 (100)	1.00
Sexual orientation				**0.03**
Heterosexual	86 (61)	33 (73)	53 (55)	
Homosexual	46 (33)	8 (18)	38 (40)	
Bisexual	9 (6)	4 (9)	5 (5)	
Number of different sexual partner within the past 3 months (median, range)	1 (0–14)	1 (0–3)	1 (0–14)	0.39
Number of new sexual partner within the past 3 months (median, range)	0 (0–11)	0 (0–3)	0 (0–11)	0.63
Using condom with vaginal sex consistently^f^	30/97 (31)	13/37 (35)	17/60 (28)	0.48
Using condom with anal sex consistently^f^	17/71 (24)	6/17 (35)	11/54 (20)	0.21
Using condom with oral sex consistently^f^	10/91 (11)	3/23 (13)	7/68 (10)	0.72
Time since last unprotected sex (months, median, range)	2 (1–72)	3 (1–48)	1 (1–72)	0.38
Ever injected drug with needle	2 (1)	1 (2)	1 (1)	0.54
Ever had tattoo or piercing	54 (38)	15 (33)	39 (41)	0.41
Ever exchanged sex for money or drugs	0 (0)	0 (0)	0 (0)	–
Having STIs within the past year				0.34
Yes	3 (2)	2 (4)	1 (1)	
Not sure	28 (20)	10 (22)	18 (19)	
Sexual partner had STIs within the past year				0.36
Yes	1 (1)	0 (0)	1 (1)	
Not sure	45 (32)	11 (24)	34 (35)	
Sexual partner exchanged sex for money or drugs within the past 30 months				0.71
Yes	2 (1)	1 (2)	1 (1)	
Not sure	30 (21)	8 (18)	22 (23)	
Sexual partner injected drug with needle within the past 3 months				1.00
Yes	0 (0)	0 (0)	0 (0)	
Not sure	27 (19)	8 (18)	19 (20)	

NOTE

Data are in numbers (%) unless otherwise indicated

^a^ Comparison between students with HIV-positive and HIV-negative test results

^b^ Included Faculty of Medicine and Faculty of Medical Technology

^c^ Included Faculty of Science and Faculty of Engineering

^d^ Included Faculty of Laws and Faculty of Journalism and Mass Communication

^e^ Based on number of correct answers to 12 statements about HIV transmission

^f^ The denominators were those who had each type of sexual activity

*STI* sexually-transmitted infection

The *P* values in bold represent statistical significance

### Characteristics associated with HIV infection

Among the 494 students undergoing HIV test, 4 (0.8 %) were HIV-infected. All of these 4 students were newly diagnosed with HIV infection. Of these 4 HIV-infected students, 4 (100 %) were male, 3 (75 %) were homosexual, 3 (75 %) had moderate to high HIV risk and 1 (25 %) had low risk perception. All of these 4 students were contacted and informed the result within the median time of 8 days (range 3–13 days). Comparing between HIV-positive and HIV-negative students (Table 5), HIV-positive students were more-likely to be homosexual, have higher median number of different sexual partner within the past 3 months and have ever had injected drug with needle. In the multivariable logistic regression analysis, characteristics independently associated with HIV positivity were being homosexual men (aOR 12.75; 95 % CI 1.30–125.06: *P* = 0.03). Of the 4 HIV-infected students, only 2 (50 %) showed up for continuity care within 3 months after the diagnosis while the other 2 could not be further contacted after the first result notification call.

**Table 5:** Characteristics of the 494 students undergoing HIV counseling and testing categorized by the HIV test result

Characteristics	All (*N* = 494)	HIV-positive (*N* = 4)	HIV-negative (*N* = 490)	*P* ^*a*^
Demographics				
Sex				0.21
Male	277 (56)	4 (0)	273 (56)	
Female	215 (44)	0 (0)	215 (44)	
Transgender	2 (0.4)	0 (0)	2 (0.4)	
Age (years, median, range)	20 (17–35)	21 (19–22)	20 (17–35)	0.84
Faculty				0.24
Health Science^b^	145 (29)	0 (0)	145 (30)	
Science^c^	168 (34)	1 (25)	167 (34)	
Social Science^d^	181 (37)	3 (75)	178 (36)	
Monthly household income ($US, median, range)	1515 (121–90,909)	1712 (1515–2970)	1515 (121–90,909)	0.53
HIV transmission knowledge score^e^ (median, range)	11 (6–12)	11 (10–11)	11 (6–12)	0.75
HIV risk charactertistics				
Prior HIV test	48 (10)	1 (25)	47 (10)	0.34
Number of prior HIV test (median, range)	1 (1–6)	0 (0–1)	1 (1–6)	0.53
Ever had sexual intercourse	202 (41)	3 (75)	199 (41)	0.31
Sexual orientation				**0.001**
Heterosexual	416 (84)	1 (25)	415 (85)	
Homosexual	65 (13)	3 (75)	62 (13)	
Bisexual	13 (3)	0 (0)	13 (3)	
Number of different sexual partner within the past 3 months (median, range)	1 (0–14)	2 (1–14)	1 (0–10)	**0.04**
Number of new sexual partner within the past 3 months (median, range)	0 (0–11)	1 (0–11)	0 (0–8)	0.14
Using condom with vaginal sex consistently^f^	58/147 (40)	–	58/147 (40)	–
Using condom with anal sex consistently^f^	31/90 (34)	1/3 (33)	30/87 (35)	0.97
Using condom with oral sex consistently^f^	19/104 (18)	0/2 (0)	19/102 (19)	0.50
Time since last unprotected sex (months, median, range)	2 (1–72)	3 (1–24)	2 (1–72)	0.74
Ever injected drug with needle	3 (0.6)	1 (25)	2 (0.4)	**<0.001**
Ever had tattoo or piercing	184 (37)	1 (25)	183 (37)	1.00
Having STIs within the past year				0.80
Yes	3 (0.6)	0 (0)	3 (0.6)	
Not sure	28 (6)	0 (0)	28 (6)	
Sexual partner had STIs within the past year				0.64
Yes	1 (0.2)	0 (0)	1 (0.2)	
Not sure	45 (9)	0 (0)	45 (9)	
Sexual partner exchanged sex for money or drugs within the past 30 months				0.75
Yes	2 (0.4)	0 (0)	2 (0.4)	
Not sure	30 (6)	0 (0)	30 (6)	
Sexual partner injected drug with needle within the past 3 months				1.00
Yes	0 (0)	0 (0)	0 (0)	
Not sure	27 (6)	0 (0)	27 (6)	
Self-perception of HIV risk				0.09
No to low risk	342 (69)	1 (25)	341 (70)	
Moderate to high risk	152 (31)	3 (75)	149 (30)	
Actual HIV risk				**0.006**
No to low risk	353 (72)	0 (0)	353 (79)	
Moderate to high risk	141 (29)	4 (100)	137 (18)	
False perception of low HIV risk	45 (9)	1 (25)	44 (9)	0.32
Able to be contacted for HIV test result	484 (98)	4 (100)	480 (98)	1.00
Time to inform HIV test result (days, median, range)	8 (2–44)	8 (3–13)	8 (2–44)	0.71

NOTE

Data are in numbers (%) unless otherwise indicated

^a^Comparison between students with HIV-positive and HIV-negative test results

^b^Included Faculty of Medicine and Faculty of Medical Technology

^c^Included Faculty of Science and Faculty of Engineering

^d^Included Faculty of Laws and Faculty of Journalism and Mass Communication

^e^Based on number of correct answers to 12 statements about HIV transmission

^f^The denominators were those who had each type of sexual activity

*STI* sexually-transmitted infection

The *P* values in bold represent statistical significance

## Discussion

Our study findings have important implications for implementation of HTC program among university students. First, our outreach HIV prevention program was successful in attracting many students to participate in during the 2-year study period. This provided opportunities to increase awareness about HIV/AIDS prevention among the students and to educate them on specific issues about risk perception and risk behaviors reduction. Second, to our knowledge, this study was the first to assess the actual rate of HTC acceptance during the outreach program for university students. This “actual” rate of HTC acceptance was significantly lower than the rates of “willingness” to have HTC assessed through a study questionnaire in previous studies (63–72 %) [6, 7]. This implies that students who indicate that they are willing to have HTC may not really have HTC in their real lives, which is an important barrier for HIV transmission prevention. Third, we identified several factors associated with no HTC acceptance among the students. Female were less-likely to accept HTC than male possibly due to perception of lower risk, being more afraid of blood/pain/needles when getting tested and stigmatization of having HIV tests. The other factors associated with no HTC acceptance included lower HIV transmission knowledge score, younger age and being students from non-health science faculties. These factors are relevant to less knowledgeable and experience about HIV/AIDS and were previously reported to be associated with no previous HIV testing and/or no willingness to have HTC among university students in other settings [6–10]. Altogether, our findings suggest the importance of HTC acceptance assessment and interventions to improve HTC acceptance including education about HIV/AIDS and HTC for the students, use of cost-effective non-blood and painless HIV testing and strategies to reduce HIV stigmatization.

The incorporation of validated HIV risk perception assessment in our outreach program was unique. This enabled the HIV counselors to identify students with false perception of low HIV risk and provide education to correct their risk perception. Based on the study risk categorization tool, 29 % of the students undergoing HTC had moderate to high risk for HIV acquisition; 69, 76 and 89 % used condom inconsistently for vaginal, anal and oral sex, respectively; 22 % of the students used to have or were not sure that they had STIs in the past year, and about one fifth to one third were not sure about their sex partners’ risk behaviors. Although the proportion of students with moderate to high HIV risk was not as high as other high-risk populations including individuals attending an STI clinic (83 %) [5], participants attending a black gay pride event (67 %) [11] and men who have sex with men (MSM) attending a gay sauna (58 %) [our unpublished data], false perception of low HIV risk was prevalent. Being heterosexual was the independent factor associated with false perception of low HIV risk in this study consistent with reports from studies among STI clinic attenders and AIDS Help-Line users [12, 13]. This may be explained by the belief that associates HIV risk with particular risk-recognized groups, i.e., MSM, injecting drug users and commercial sex workers rather than their risk behaviors [5, 14, 15]. The underlying mechanisms for false perception of low HIV risk among the students despite the high level of knowledge may include their risk assessment in light of remote or past low-risk behaviors despite current high-risk behaviors, optimistic bias that the risk behaviors they engaged in were at no or low-risk, denial or suppression mechanism and bias that they did not use facts in their judgment about risk perception but used certain cues inherent in the questions asked [16].

Previous studies demonstrated that 10–55 % of individuals undergoing blood-based HTC did not return for HIV results [17, 18]. However, our study demonstrated the high rates of result notification (98 % for all students and 100 % for HIV-infected students). The higher rate of result notification in our study compared to those in the previous studies could be due to the use of HIV counselor-initiated confidential telephone contact with 2 weekly-follow-up calls rather than having the participants calling in for the results. Although the rapid HIV testing and result notification within 1 h is ideal strategy, these findings suggest that our test result notification protocol may be used in resource-limited settings where the rapid testing is not available. The lower rate of HIV infection among the university students compare to that of Thai general population is due to the overall lower risk of HIV acquisition among the students. Being homosexual men was independently associated with HIV positivity. This finding was consistent with reported high incidence of HIV infection among Thai MSM [19]. Despite being notified with the test result and receiving post-test counseling, only 50 % of HIV-infected students had engaged in HIV continuity care. Interventions such as point-of-care CD4 testing at the time of HTC [20], on-site HIV care providers to perform focused opportunistic infection screening and immediate antiretroviral therapy initiation if eligible, use of non-cash financial incentives for linkage to care [21], more emphasis and clear message to the students on the importance of linkage to care, antiretroviral therapy adherence and care retention, addressing plan for long-term care based on the student’s medical coverage, preference and financial status may improve the students’ linkage to care.

There were limitations in this study. First, the results may have limited generalizability to university students from a large public university. However, the findings of challenges in HTC acceptance and linkage to care, the importance of HIV risk perception assessment and targeted education based of the risk perception and the result notification protocol should be applicable to implementation of HTC in other settings. Second, the face-to-face interview during the pre- and post-test counseling might impact the disclosure of HIV risks and risk behaviors of the participants. However, this limitation was minimized since the counseling was done in a private room and the counselors were well-trained to build trust between the students and them. Lastly, the small number of HIV-infected students may limit identification of other factors associated with HIV infection. Nonetheless, the result can be a reference rate of HIV infection among university students in our setting.

## Conclusions

Implementation of an outreach HIV prevention program incorporating HTC was feasible and provided opportunities to educate the students about HIV transmission, risk perception and risk behavior reduction. HIV risk perception assessment along with interventions to correct risk perception are necessary and should be included in the program. Rapid HIV testing and result notification, point-of-care CD4 testing and HIV care and educational interventions in both healthcare and community settings are needed to increase access to HIV testing and successful linkage to care.

## Abbreviations

AIDS, acquired immune deficiency syndrome; aOR, adjusted odds ratio; CI, confidence interval; HIV, human immunodeficiency virus; HTC, HIV testing and counseling; MSM, men who have sex with men; STIs, sexually-transmitted infections

## References

[1] Averting HIV and AIDS. HIV and AIDS in Thailand. Available at http://www.avert.org/professionals/hiv-around-world/asia-pacific/thailand. Accessed 10 Mar 2016.

[2] Ministry of Public Health of Thailand. HIV/AIDS status report. Available at http://www.boe.moph.go.th/files/report/20110401_19956923.pdf. Accessed 10 Mar 2016.

[3] Khawcharoenporn T, Chunloy K, Apisarnthanarak A (2015). HIV knowledge, risk perception and pre-exposure prophylaxis interest among Thai university students. Int J STD AIDS.

[4] Center for Disease Control and Prevention (CDC) (2006). Revised Recommendations for HIV Testing of Adults, Adolescents, and Pregnant Women in Health-Care Settings. MMWR.

[5] Khawcharoenporn T, Kendrick S, Smith K (2012). HIV risk perception and preexposure prophylaxis interest among a heterosexual population visiting a sexually transmitted infection clinic. AIDS Patient Care STDs.

[6] Abiodun O, Sotunsa J, Ani F, Jaiyesimi E (2014). Knowledge of HIV/AIDS and predictors of uptake of HIV counseling and testing among undergraduate students of a privately owned university in Nigeria. BMC Res Notes.

[7] Oppong Asante K (2013). HIV/AIDS knowledge and uptake of HIV counseling and testing among undergraduate private university students in Accra, Ghana. Reprod Health.

[8] Fikadie G, Bedimo M, Alamrew Z (2014). Prevalence of voluntary counseling and testing utilization and its associated factors among Bahirdar University students. Adv Prev Med.

[9] Addis Z, Yalew A, Shiferaw Y, Alemu A, Birhan W, Mathewose B (2013). Knowledge, attitude and practice towards voluntary counseling and testing among university students in North West Ethiopia: a cross sectional study. BMC Public Health.

[10] Norman LR, Gebre Y (2005). Prevalence and correlates of HIV testing: An analysis of university students in Jamaica. J Int AIDS Soc.

[11] Khawcharoenporn T, Kendrick S, Smith K. Does HIV risk perception affect condom use and pre-exposure prophylaxis (PrEP) interest? An examination of sexually transmitted infection clinic attendees and Black Gay Pride Event participants. In: the 6th International AIDS Society Conference on HIV Pathogenesis, Treatment and Prevention. July 17-20, 2011. Rome, Italy. Abstract 656

[12] James NJ, Gillies PA, Bignell CJ (1991). AIDS-related risk perception and sexual behaviour among sexually transmitted disease clinic attenders. Int J STD AIDS.

[13] Gallo P, Colucci A, Camoni L, Regine V, Luzi AM, Suligoi B (2011). Social and behavioural characteristics of a sample of AIDS Help-Line users never tested for HIV in Italy. Eur J Public Health.

[14] Adimora AA, Schoenbach VJ (2002). Contextual factors and the black-white disparity in heterosexual HIV transmission. Epidemiology.

[15] Magnus M, Kuo I, Shelley K, Rawls A, Peterson J, Montanez L (2009). Risk factors driving the emergence of a generalized heterosexual HIV epidemic in Washington, District of Columbia networks at risk. AIDS.

[16] Ndugwa Kabwama S, Berg-Beckhoff G (2015). The association between HIV/AIDS-related knowledge and perception of risk for infection: a systematic review. Perspect Public Health.

[17] Sullivan PS, Lansky AL, Drake A (2004). Failure to return for HIV test results among persons at high risk for HIV infection. J Acquir Immune Defic Syndr.

[18] Hightow LB, Miller WC, Leone PA, Wohl D, Smurzynski M, Kaplan AH (2003). Failure to return for HIV posttest counseling in an STD clinic population. AIDS Educ Prev.

[19] Dokubo EK, Kim AA, Le LV, Nadol PJ, Prybylski D, Wolfe MI (2013). HIV Incidence in Asia: A review of available data and assessment of the epidemic. AIDS Rev.

[20] McNairy ML, Gachuhi AB, Lamb MR, Nuwagaba-Biribonwoha H, Burke S, Ehrenkranz P (2015). The Link4Health study to evaluate the effectiveness of a combination intervention strategy for linkage to and retention in HIV care in Swaziland: protocol for a cluster randomized trial. Implement Sci.

[21] Newman PA, Lee SJ, Roungprakhon S, Tepjan S (2012). Demographic and behavioral correlates of HIV risk among men and transgender women recruited from gay entertainment venues and community-based organizations in Thailand: implications for HIV prevention. Prev Sci.

